# The Impact of Diet on Hidradenitis Suppurativa Severity: A Cross-Sectional Case–Control Study

**DOI:** 10.3390/medicina60122107

**Published:** 2024-12-23

**Authors:** Ferhan Kesik, Sibel Dogan-Gunaydin, Mehmet Fisunoglu

**Affiliations:** 1 Department of Nutrition and Dietetics, Faculty of Health Sciences, Gaziantep University, 27310 Gaziantep, Turkey; 2 Department of Dermatology and Venereology, School of Medicine, Hacettepe University, 06100 Ankara, Turkey; sibel.dogan@hacettepe.edu.tr; 3 Department of Nutrition and Dietetics, Faculty of Health Sciences, Hacettepe University, 06100 Ankara, Turkey; fisunogl@hacettepe.edu.tr

**Keywords:** hidradenitis suppurativa, diet, nutrition, Mediterranean, glycemic index, nightshades, dairy, food

## Abstract

*Background and Objectives:* Hidradenitis suppurativa (HS) is a chronic inflammatory skin disease primarily affecting hair follicles, characterized by painful nodules, abscesses, and sinus tract formation. Recent evidence suggests that weight management and nutritional factors may influence HS symptoms. This cross-sectional case–control study aimed to assess the impact of body composition and nutritional factors on HS severity. *Materials and Methods:* We included 50 patients with HS and 50 matched controls comparable in body mass index (BMI), sex, and age. The data collected included 3-day food records, a food frequency questionnaire, 24 h physical activity records, Mediterranean Diet Adherence Screener (MEDAS) scores, body composition, and anthropometric measurements. The macronutrient and micronutrient intake, as well as dietary glycemic index (GI), were analyzed. HS severity was assessed using the Hurley staging system and the International Hidradenitis Suppurativa Severity Score System (IHS4). *Results:* HS patients exhibited significantly lower adherence to the Mediterranean diet and a higher dietary GI compared to controls. Their micronutrient intake was also reduced, while the consumption of junk food, dairy products, and nightshade vegetables was more frequent. The MEDAS scores and physical activity levels were negatively correlated with the IHS4 scores, while higher anthropometric measurements, dietary energy, protein, total fat, and GI showed positive correlations. The Hurley stage was negatively correlated with the MEDAS scores and positively correlated with the GI and visceral fat. In multiple regression analysis, the MEDAS score emerged as the primary variable associated with disease severity. *Conclusions:* These findings suggest that an increased adherence to the Mediterranean diet, intake of food with a lower GI, and maintaining an ideal body weight may positively affect HS management. Long-term studies are warranted to corroborate our findings.

## 1. Introduction

Hidradenitis suppurativa (HS), also known as acne inversa, is a chronic non-contagious inflammatory skin disorder characterized by subcutaneous nodules, abscesses, sinus tracts, and scarring [1,2]. HS primarily affects body regions rich in apocrine glands, such as the axillary, inguinal, gluteal, and inframammary areas [3]. The estimated prevalence of HS in the general population ranges from 0.05% to 4.0%, with diagnostic delays of up to 7 years due to non-specific symptoms and frequent misdiagnoses [4]. The exact etiology and pathogenesis of HS remain unclear [5]. The management of HS includes a range of therapeutic strategies, such as antibiotics to mitigate inflammation, novel biologic agents targeting specific cytokines implicated in HS pathogenesis, and surgical interventions for advanced cases [6]. Despite the availability of optimal treatments, the possibility of therapeutic resistance and lesion recurrence underscores the importance of integrating lifestyle modifications to enhance the treatment outcomes [7].

For patients with HS, lifestyle modifications that target the diet, physical activity, and weight management may help reduce disease severity [8]. Diets thought to alleviate HS symptoms typically focus on an increased intake of fruits and vegetables while reducing processed foods, refined carbohydrates, and sugars [8]. As such, the Mediterranean diet has gained attention for its health benefits and potential to reduce inflammation [9]. This diet emphasizes the consumption of extra-virgin olive oil, vegetables, fruits, grains, legumes, and nuts, along with moderate amounts of fish, dairy products, and red wine, while limiting eggs and sweets [10]. The Mediterranean diet’s anti-inflammatory effects are attributed to its high content of fiber, polyphenols, carotenoids, and omega-3 fatty acids [10,11,12]. In contrast, the Western diet, which is rich in dairy products and refined carbohydrates, may cause hair follicle blockage. Dairy components such as casein and whey increase IGF-1 and insulin levels, respectively, thereby enhancing androgen receptor activity and exacerbating HS symptoms. Foods with a high glycemic index (GI), such as junk food (e.g., cakes, chocolate, cookies, and potato chips) and bakery products, may also worsen HS severity by inducing hyperinsulinemia and hyperandrogenism [9].

Research has shown that patients with HS exhibit higher insulin resistance than age- and sex-matched controls, even after adjusting for body mass index [13]. The association between high-glycemic-index foods and HS is further supported by the presence of anti-CEL antibodies, which are linked to advanced glycation end products (AGEs) in patients with HS [14]. AGEs bind to specific receptors, triggering the release of pro-inflammatory cytokines, such as IL-1β and TNF-α, which contribute to follicular hyperkeratosis, inflammation, and fibrosis [9]. As a result, a low-glycemic-index diet has been proposed as potentially beneficial for HS by reducing insulin spikes and androgen receptor activation [9,15].

Additionally, elimination diets that exclude nightshade vegetables (Solanaceae) have been suggested to relieve HS symptoms, possibly due to their glycoalkaloid content, including alpha-tomatine, capsaicin, and solanine [4]. However, evidence supporting specific dietary interventions for HS remains limited [9]. The 2018 British Association of Dermatologists’ guidelines on HS management and the 2019 U.S. and Canadian clinical guidelines have both concluded that there is insufficient high-quality evidence to recommend any specific diet or nutrient supplementation for HS [16,17]. Therefore, further comprehensive research is needed to better understand the role of diet in HS management [18].

Thus, this study aimed to comprehensively evaluate the nutritional status of patients with HS and investigate the relationship between HS’s clinical severity and modifiable lifestyle factors beyond diet, including body composition, anthropometric measurements, and physical activity levels. By clarifying the potential impact of these modifiable factors, our goal was to inform the development of more effective management strategies for patients with HS.

## 2. Materials and Methods

This cross-sectional, case–control study was conducted at the Dermatology and Venereology outpatient clinics of Hacettepe University Hospitals, Turkey, between January 2023 and January 2024. Approval for the study was granted by the Institutional Review Board of Hacettepe University (16 March 2021; no. 202/06-22). The study was conducted in accordance with the ethical principles laid out in the Declaration of Helsinki. The nature and scope the study were explained to all participants, and written informed consent was obtained.

### 2.1. Study Sample

The study included 50 patients diagnosed with HS and 50 controls, matched for body mass index (BMI), sex, and age. The control group consisted of individuals who attended the dermatology department due to skin disorders other than HS, such as xerosis cutis, and who did not have any chronic inflammatory diseases. Individuals in both groups were excluded if they were using multivitamin–mineral or antioxidant supplements, receiving any medical nutrition therapy, or pregnant or lactating. Data collected for both groups included sociodemographic characteristics, 3-day food records, food intake frequency for specific food items (dairy products, nightshade vegetables, junk foods, and bakery products), 24 h physical activity logs, body composition, anthropometric measurements, and Mediterranean Diet Adherence Screener (MEDAS) scores. The macronutrient and micronutrient intake, as well as the dietary GI, were also calculated. The severity of HS was assessed in the patient group.

### 2.2. Three-Day Food Records

A 3-day food diary was obtained from each participant to document the daily intake. These records were examined for macronutrient and micronutrient content using the Nutrition Information Systems Software. The food items consumed were entered into the program in grams, enabling the calculation of macronutrient and micronutrient content for each item.

### 2.3. Dietary Glycemic Index

The glycemic index (GI) was determined using each participant’s 3-day food records. The average GI of the diet was calculated based on the international glycemic index table by Atkinson et al. [19].

### 2.4. Food Frequency Questionnaire

This questionnaire was used to assess the quantity and frequency of foods identified in the literature as potentially associated with HS symptoms [4]. These foods included dairy products (e.g., milk, yogurt, cheese, and kefir), nightshade vegetables (e.g., potatoes, tomatoes, eggplants, and peppers), refined sugar, junk foods (e.g., cakes, chocolate, cookies, and potato chips), and bakery products (e.g., bread, bagels, pastries, and pizza). The frequency of consumption was categorized as frequent (daily or 5–6 times per week), moderate (1–4 times per week), or rare (less than once per week).

### 2.5. Adherence to the Mediterranean Diet

The adherence to the Mediterranean diet was assessed using the 14-item Mediterranean Diet Adherence Scale (MEDAS) developed by Schröder et al. [20]. The scale includes questions on core components of the Mediterranean diet, such as the consumption of extra-virgin olive oil, vegetables, fruits, oilseeds, and fish. A total MEDAS score ranging from 0 to 5 is classified as low adherence, a score between 6 and 9 indicates moderate adherence, and a score of ≥10 represents high adherence. Higher scores indicate greater adherence to the Mediterranean diet, while lower scores reflect non-adherence.

### 2.6. Body Composition and Anthropometric Measurements

Body weight, height, waist circumference, and hip circumference were measured for all the participants. The BMI and waist-to-hip ratio were subsequently calculated. Body composition was analyzed using a bioelectrical impedance analyzer (Tanita MC-980, Tanita Corporation, Tokyo, Japan).

### 2.7. Physical Activity Level

Each participant’s physical activity level (PAL) was assessed through a 24 h physical activity record. PAL values between 1.40 and 1.69 were considered sedentary, 1.70 to 1.99 was classified as moderate, and values of 2.0 or higher were categorized as vigorous physical activity, based on the WHO criteria [21].

### 2.8. Clinical Severity Assessment

For each patient, the clinical severity of HS was determined using the Hurley system and the International Hidradenitis Suppurativa Severity Scoring System (IHS4). The Hurley system, developed in 1989 and still widely used, qualitatively assesses HS in three stages based on the presence of nodules, abscesses, sinus tracts, and scarring [22]. The IHS4, a more recent tool, provides a quantitative assessment of disease severity based on the number of nodules, abscesses, fistulas, and sinuses. In this scale, scores of 3 or below indicate mild HS, scores of 4–10 indicate moderate HS, and scores of 11 or higher indicate severe HS [23]. Additionally, data regarding the age at which the disease manifested, the age at which a diagnosis was given, the length of time between the onset of symptoms and the diagnosis, and the overall duration of the disease were gathered.

### 2.9. Statistical Analysis

The data were analyzed using SPSS version 22 (IBM Corp., Armonk, NY, USA). Qualitative data are presented as the frequency and percentage, while quantitative data are shown as the mean and standard deviation. The Shapiro–Wilk test was used to assess the normality of the data distribution. Group comparisons were made using the Chi-square test for qualitative data, an independent samples *t*-test for normally distributed data, and the Mann–Whitney U test for non-normally distributed data. Pearson correlation analysis was used to examine relationships between normally distributed variables, while Spearman correlation analysis was employed for non-normally distributed variables. Additionally, linear regression analysis was used to identify predictive relationships. A significance level of α = 0.05 was used for all tests.

## 3. Results

No significant differences were observed between the two groups in terms of age, sex, physical activity level (PAL), anthropometric measurements, or body composition. Among the HS patients, 18% were classified as stage I, 28% as stage II, and 54% as stage III according to the Hurley staging system. Based on IHS4 scoring, 8% of the patients were categorized as having mild HS, 30% as moderate, and 62% as severe. The general characteristics of the participants are shown in Table 1.

A 3-day food consumption record was collected from both the HS patients and the controls. The mean dietary GI was significantly higher in the HS group than in the control group. Additionally, the food consumption records revealed that the HS group had significantly lower intakes of vitamin C and iron compared to the control group. The dietary GI and daily macro- and micronutrient intakes, based on the 3-day food records for both groups, are presented in Table 2.

The frequency of cheese and yogurt consumption, as well as junk food consumption, was significantly higher in HS patients compared to the control group. Furthermore, the daily intake of yogurt, peppers, nightshade vegetables (including potatoes, eggplants, tomatoes, and peppers), and junk food was significantly greater for HS patients than for the control group. Table 3 provides a comparison of the frequency and daily consumption quantities of these foods between the two groups.

The data from the MEDAS questionnaire indicated that the mean adherence score to the Mediterranean diet was 4.30 ± 1.78 for the HS group and 6.04 ± 1.92 for the control group (*p* = 0.000). In this study, the HS group demonstrated low adherence to the Mediterranean diet, while the control group exhibited moderate adherence. A significant majority of the patients with HS (86%, 43/50) showed low adherence to the Mediterranean diet, while 14% (7/50) demonstrated moderate adherence, and none (0%) achieved high adherence. In contrast, 56% (28/50) of the control group exhibited low adherence, 34% (17/50) demonstrated moderate adherence, and 10% (5/50) showed high adherence.

The highest frequency of affirmative responses was found for the use of extra-virgin olive oil as the primary culinary lipid: 60.0% (30/50) for HS patients and 72.0% (36/50) for controls. Conversely, the lowest frequency of affirmative responses was observed for the consumption of wine at ≥7 glasses per week (0% for both groups). Although some individuals in both groups consumed alcohol, none reported regularly consuming wine. Table 4 presents the MEDAS scores and response rates for dietary items listed in the MEDAS questionnaire.

Compared to the controls, the patients with HS showed significant differences in the consumption of extra-virgin olive oil, red meats, processed meats, butter, cream, margarine, carbonated beverages, sugar-sweetened beverages, and commercial pastries such as cookies or cake (*p* < 0.05). Additionally, specific dietary habits were associated with lower IHS4 scores, including using extra-virgin olive oil as the primary culinary lipid, consuming more than four tablespoons of extra-virgin olive oil daily, consuming three or more servings of nuts weekly, and limiting the intake of commercial pastries to fewer than two servings per day (*p* < 0.05). Furthermore, both the use of extra-virgin olive oil as the primary cooking fat and a daily intake of more than four tablespoons were associated with a reduction in the Hurley staging, as assessed by the Hurley staging system (*p* < 0.05).

Correlation analyses of clinical severity were conducted using both the Hurley staging and IHS4 scores. The results showed negative correlations between the IHS4 score and PAL, MEDAS score, and daily vitamin C intake. In contrast, positive correlations were observed between IHS4 scores and dietary glycemic index, daily intake of energy, protein, and fat, as well as BMI, waist circumference, waist-to-hip ratio, fat mass, and visceral fat in patients with HS.

Similarly, daily vitamin C intake, zinc intake, and the MEDAS score were negatively correlated with Hurley staging, while the dietary glycemic index and visceral fat were positively correlated with Hurley staging. The daily consumption of refined sugar, junk food, and bakery products was positively correlated with both Hurley staging and IHS4 scores. Table 5 shows the correlations between Hurley staging, IHS4 scores, and variables such as PAL, anthropometric measurements, BIA measurements, nutritional factors, MEDAS scores, and the daily consumption of specific foods.

Univariate regression analysis identified the MEDAS score and PAL as negative predictors of HS disease severity, while dietary glycemic index and BMI emerged as positive predictors. In multivariate models, however, only the MEDAS score consistently emerged as a significant predictor of HS disease severity for both Hurley staging and IHS4 scores (Table 6).

## 4. Discussion

This study is one of the most comprehensive investigations to date that has aimed to elucidate the impact of nutritional factors and adherence to the Mediterranean diet on the severity of HS. The primary findings of this cross-sectional case–control study indicate significant associations between the clinical severity of HS and several modifiable lifestyle factors. In particular, the study found that higher IHS4 scores in HS patients were associated with lower adherence to the Mediterranean diet; dietary patterns characterized by a higher glycemic index, increased energy, higher protein and fat intake, and reduced vitamin C consumption; elevated anthropometric measurements (e.g., body mass index, waist-to-hip ratio); increased BIA parameters (e.g., visceral fat, body fat mass); and reduced physical activity levels. Similarly, Hurley staging showed an inverse association with adherence to the Mediterranean diet and dietary vitamin C and zinc intake while exhibiting a positive association with visceral fat.

Obesity is widely recognized as a significant factor that exacerbates HS progression [1], and numerous studies have reported a positive correlation between higher BMI and greater disease severity in patients with HS [4,17,24,25]. In line with those reports, we found a positive correlation between BMI and IHS4 scores; however, no significant association was observed with Hurley staging. Additionally, the key indicators of abdominal obesity—waist circumference, waist-to-hip ratio, and visceral fat—were significantly associated with IHS4 scores.

Adherence to the Mediterranean diet was another key focus of this study. We found that patients with HS had a significantly lower adherence to the Mediterranean diet compared to the control group, and disease severity was negatively correlated with adherence. Specifically, a high intake of olive oil and nuts, as well as the minimal consumption of commercial pastries, were most strongly associated with reduced disease severity. According to our findings, so far, only three studies have examined the effect of the Mediterranean diet on HS disease severity [12,26,27]. Barrea et al. [26] reported that greater compliance with the Mediterranean diet was linked to lower Sartorius scores. Similarly, Lorite-Fuentes et al. [27] found that higher adherence to the Mediterranean diet was correlated with lower self-reported Hurley and IHS4 scores. However, Velluzzi et al. [12] did not identify any notable correlations between disease severity and dietary factors. Nevertheless, their HS patients demonstrated lower adherence to the Mediterranean diet and more unfavorable anthropometric measurements compared to the control group [12].

Following the Mediterranean diet, another dietary model that stands out for its impact on HS symptoms is the ketogenic diet. A 28-day pilot study by Verde et al. [28] demonstrated that obese women with HS who adhered to a very low-calorie ketogenic diet (VLCKD) experienced significant reductions in disease severity, systemic inflammatory markers (including trimethylamine-N-oxide [TMAO], oxidized low-density lipoprotein [ox-LDL], and derivatives of reactive oxygen metabolites [dROMs]), as well as improvements in anthropometric measures such as BMI and fat mass. The dietary regimen was carefully designed to be low in energy, was supplemented with essential vitamins and minerals to prevent deficiencies, and incorporated high-biological-value protein sources, including egg, pea, soy, and whey proteins. While these findings highlight the potential benefits of VLCKD for HS management, randomized longitudinal studies are needed to further evaluate the efficacy and safety of this dietary approach [28].

Our analysis revealed that higher intakes of energy, protein, and fat were associated with elevated IHS4 scores, and a positive correlation was observed between the consumption of refined sugar, junk food, and bakery products, and increased disease severity. These findings align with previous research suggesting that diets rich in fat and sugar may exacerbate HS symptoms [29,30,31]. The Western diet, characterized by high-glycemic index foods, red meat, dairy products, and egg protein, has similarly been implicated in the worsening of HS severity [32]. Furthermore, studies have indicated that patients with HS have greater insulin resistance than age- and sex-matched controls, even after adjusting for BMI [13]. In light of these findings, a low-glycemic index diet has been proposed as a potentially beneficial dietary approach for managing HS [15]. In our study, the intake of a high-GI diet was significantly more common among patients with HS than controls, and higher GI was correlated with greater disease severity.

Dairy products may have adverse effects on HS [33]. Components of cow milk, such as casein, whey proteins, hormones, and growth factors, can elevate androgen receptor activity by increasing insulin and IGF-1 levels, leading to hyperandrogenism and potentially exacerbating HS symptoms [33]. Additionally, the amino acid leucine, present in dairy products, may contribute to HS by activating the mTOR pathway [32]. Notably, many patients with HS who eliminated dairy products from their diet reported improvements in their symptoms [34]. Furthermore, certain components of nightshade vegetables, such as alkaloids, alpha-tomatine, and capsaicin, are thought to affect HS progression negatively [4]. Dempsey et al. [30] reported that 75.8% of the 242 patients in their study modified at least one component of their diet, most commonly by reducing gluten, dairy products, refined sugars, tomatoes, and alcohol. However, these dietary modifications improved symptoms in some patients while exacerbating them in others. Another study by Fernandez et al. [29] reported that 32.6% of patients with HS identified specific foods—particularly sweets, bread/pasta, dairy products, and high-fat foods—as aggravating their condition. In our study, we observed a positive correlation between cheese consumption and Hurley staging, indicating that a higher cheese intake may be associated with greater disease severity. While dairy elimination diets have shown benefits in some cases, they should be reserved for HS patients with confirmed sensitivities or intolerances or evaluated on an individual basis [35].

A review of the macro- and micronutrient intake from the 3-day food records for both the HS and control groups showed lower intakes of vitamin C and iron in patients with HS compared to the controls. Daily vitamin C intake was negatively correlated with both IHS4 scores and Hurley staging, while zinc intake was negatively correlated with Hurley staging alone. Vitamin C is known to play a therapeutic role in skin health by stabilizing collagen, regulating ceramide levels, promoting wound healing, and exerting antioxidant effects [36]. Zinc, on the other hand, is crucial in regulating both innate and adaptive immune responses, and is involved in metabolic and hormonal pathways [9]. Its essential role in immune regulation and wound healing suggests its involvement in the pathogenesis of inflammatory skin diseases such as HS, which are driven by immune dysregulation [9]. The lower serum zinc levels observed in patients with HS, compared to controls, suggest that zinc supplementation could serve as a beneficial adjunctive therapy [37].

We also observed a significant negative correlation between PAL and IHS4 scores, although no significant association was found with Hurley staging. This finding is consistent with the results of Lorite-Fuentes et al. [27], who noted that vigorous physical activity was associated with reduced IHS4 scores. While physical activity can support weight management and reduce inflammation [8], many patients face challenges in maintaining regular physical activity due to painful lesions and discomfort with movement [38]. Factors such as increased body temperature, sweating, and friction during exercise can also aggravate symptoms, discouraging patients from engaging in sports [38].

The findings of this study highlight the potential role of dietary strategies in managing HS; however, their integration into clinical practice may not be straightforward. A multidisciplinary approach involving dermatologists and dietitians can support the development of individualized dietary interventions. Nonetheless, future longitudinal studies are needed to validate these findings and establish robust dietary guidelines for HS management.

This study has several limitations, including its cross-sectional design, which precludes the establishment of causal relationships. Additionally, IGF-1 and insulin levels were not investigated, which could have provided further insights into the impact of high-glycemic index foods and dairy products on the disease severity. The study was conducted in a single geographic region, which may limit the generalizability of the findings to populations with different cultural and dietary patterns. However, the study’s strengths include the matched groups for age, sex, and BMI and its contribution to the relatively limited literature on the relationship between HS and nutrition.

## 5. Conclusions

Implementing practical lifestyle changes, such as adherence to the Mediterranean diet, maintaining a low-glycemic index diet, and sustaining an ideal body weight, may be effective strategies for reducing the severity of hidradenitis suppurativa. Therefore, incorporating dietary considerations into the treatment plan may be beneficial for managing the disease. Long-term studies are warranted to corroborate our findings.

## Figures and Tables

**Table 1:** General characteristics of the participants.

	HS Patients *n* = 50	Controls *n* = 50	*p*-Value
Age (years)	34.06 ± 11.36	34.16 ± 9.55	0.702
Sex			
Female	20	20	1.00
Male	30	30	
Physical Activity			
PAL	1.56 ± 0.062	1.58 ± 0.081	0.266
Anthropometric Measurements			
Height (m)	172.12 ± 8.62	171.84 ± 9.07	0.874
Weight (kg)	83.61 ± 20.20	80.29 ± 13.92	0.605
BMI (kg/m^2^)	27.99 ± 6.08	27.16 ± 4.17	0.989
Waist Circumference (cm)	93.44 ± 19.96	90.46 ± 14.10	0.642
Hip Circumference (cm)	105.56 ± 11.90	106.04 ± 8.41	0.280
Waist-to-Hip Ratio	0.87 ± 0.11	0.84 ± 0.11	0.202
Body Composition			
Fat Mass (kg)	24.02 ± 11.44	21.58 ± 7.36	0.530
Fat Mass (%)	27.64 ± 8.63	26.76 ± 7.24	0.615
Visceral Fat (%)	7.60 ± 5.60	6.96 ± 3.99	0.923
Fat-Free Mass (%)	72.57 ± 8.40	73.04 ± 7.14	0.828
Muscle Mass (%)	69.07 ± 8.05	72.0 ± 10.5	0.869
Bone Mineral (%)	3.63 ± 0.43	3.66 ± 0.33	0.699
Total Body Water (%)	51.06 ± 5.97	53.22 ± 5.30	0.73
HS-Related Data			
Disease Duration (Years)	10.32 (±7.91)	-	-
Delay in Diagnosis (Years)	5.28 (±6.08)	-	-
Age at Diagnosis (Years)	29.16 (±10.36)	-	-
Age at Disease Onset	23.68 (±9.01)	-	-
Hurley Stage			
I	9 (18%)	-	-
II	14 (28%)		
III	27 (54%)		
IHS4			
Mild	4 (8%)	-	-
Moderate	15 (30%)		
Severe	31 (62%)		
Mean IHS4 Score	9.20 ± 3.42	-	-

**Table 2:** Dietary glycemic index and daily macro- and micronutrient intake.

Nutritional Factor	HS Patients *n* = 50	Controls *n* = 50	*p*-Value
	Mean ± SD	Mean ± SD	
Dietary Glycemic Index	59.96 ± 6.31	55.01 ± 5.72	**0.000**
Energy (kcal/day)	2079.85 ± 376.06	2160.74 ± 413.37	0.205
Protein (g)	82.33 ± 20.90	86.71 ± 20.66	0.170
Protein (%)	16.40 ± 3.90	16.56 ± 3.56	0.614
Fat (g)	79.56 ± 18.78	82.74 ± 21.23	0.465
Fat (%)	34.04 ± 4.07	33.98 ± 4.40	0.662
Carbohydrate (g)	250.73 ± 55.82	259.22 ± 58.84	0.659
Carbohydrate (%)	49.38 ± 6.31	49.26 ± 5.52	0.686
Fiber (g)	25.07 ± 6.97	26.99 ± 8.20	0.288
Vitamin A	1469.82 ± 1132.96	1742.79 ± 2479.21	0.804
Vitamin B12	3.64 ± 1.82	4.38 ± 4.99	0.860
Vitamin C (mg)	109.54 ± 53.65	141.43 ± 44.72	**0.001**
Iron (mg)	13.28 ± 3.08	15.16 ± 4.19	**0.039**
Zinc (mg)	11.52 ± 3.36	12.08 ± 2.71	0.197

A bolded *p*-value indicates a statistically significant difference (*p* < 0.05).

**Table 3:** Frequency and daily intake of foods.

Food	Consumption	Group		*p*-Value
		HS Patients *n* = 50	Controls *n* = 50	
Cow Milk	Frequent	8	17	0.086
	Moderate	19	12	
	Rare	23	21	
	AI *	57.58 ± 76.23	68.68 ± 68.90	0.434
Cheese	Frequent	39	36	**0.025**
	Moderate	9	4	
	Rare	2	10	
	AI *	42.34 ± 25.98	35.36 ± 22.73	0.121
Yogurt	Frequent	27	17	**0.011**
	Moderate	18	16	
	Rare	5	17	
	AI *	119.94 ± 80.58	79.12 ± 39.95	**0.007**
Kefir	Frequent	0	0	0.307
	Moderate	3	1	
	Rare	47	49	
	AI *	2.62 ± 6.94	4.00 ± 12.51	0.228
Dairy Products, Total **	AI *	222.48 ± 120.79	187.72 ± 88.11	0.133
Potatoes	Frequent	15	19	0.176
	Moderate	24	15	
	Rare	11	16	
	AI *	62.60 ± 63.88	54.10 ± 43.85	0.708
Eggplants	Frequent	1	1	0.472
	Moderate	25	31	
	Rare	24	18	
	AI *	36.62 ± 36.78	34.58 ± 29.17	0.863
Tomatoes	Frequent	41	34	0.268
	Moderate	7	12	
	Rare	2	4	
	AI *	88.24 ± 52.55	71.38 ± 49.80	0.088
Peppers	Frequent	25	32	0.215
	Moderate	19	11	
	Rare	6	7	
	AI *	49.98 ± 43.39	29.66 ± 28.74	**0.000**
Nightshade Vegetables, Total ***	AI *	237.44 ± 98.67	189.72 ± 67.42	**0.009**
Refined Sugar	Frequent	27	33	0.142
	Moderate	11	4	
	Rare	12	13	
	AI *	15.34 ± 10.04	11.98 ± 9.27	0.068
Junk Foods	Frequent	20	17	**0.039**
	Moderate	22	14	
	Rare	8	19	
	AI *	36.02 ± 26.12	15.52 ± 10.86	**0.000**
Bakery Products	Frequent	50	50	-
	Moderate	0	0	
	Rare	0	0	
	AI *	263.80 ± 104.07	253.1 ± 84.35	0.737

A bolded *p*-value indicates a statistically significant difference (*p* < 0.05). * AI: average intake (g/day). ** Dairy products, total: cow milk, cheese, yogurt, kefir. *** Nightshade vegetables, total: potatoes, tomatoes, peppers, eggplants.

**Table 4:** Response rates for the dietary items listed in the MEDAS questionnaire.

MEDAS	HS Patients *n* = 50	Controls *n* = 50	*p*-Value
	Mean ± SD	Mean ± SD	
MEDAS Score	4.30 ± 1.78	6.04 ± 1.92	**0.000**
	**%**	**%**	
Low adherence	86% (43)	56% (28)	**0.002**
Moderate adherence	14% (7)	34% (17)	
High adherence	0% (0)	10% (5)	
Primary use of extra-virgin olive oil	60.0% (30)	72.0% (36)	0.205
Extra-virgin olive oil, >4 tablespoons/day	26.0% (13)	48.0% (24)	**0.023**
Vegetables, ≥2 servings/day	34.0% (17)	30.0% (15)	0.668
Fruits, ≥3 servings/day	28.0% (14)	38.0% (19)	0.288
Red meat/processed meat, <1 serving/day	44.0% (22)	68.0% (34)	**0.016**
Butter, cream, and margarine, <1 serving/day	40.0% (20)	74.0% (37)	**0.001**
Carbonated and/or sugar-sweetened beverages, <1 serving/day	40.0% (20)	60.0% (30)	**0.046**
Wine, ≥7 glasses/week	%0 (0)	%0 (0)	-
Pulses, ≥3 servings/week	34.0% (17)	46.0% (23)	0.221
Fish/seafood, ≥3 servings/week	8.0% (4)	2.0% (1)	0.169
Commercial pastries such as cookies or cake, <2 servings/day	24.0% (12)	46.0% (23)	**0.035**
Nuts, ≥3 servings/day	40.0% (20)	58.0% (29)	0.072
Poultry rather than red meat	32.0% (16)	44.0% (22)	0.216
Olive oil vegetable sauce, ≥2 servings/day	20.0% (10)	16.0% (8)	0.603

A bolded *p*-value indicates a statistically significant difference (*p* < 0.05).

**Table 5:** Correlations with the severity of hidradenitis suppurativa.

Variable	Hurley Staging		IHS4 Score	
	r	*p*-Value	r	*p*-Value
Physical Activity				
Physical Activity Level (PAL)	−0.092	0.523	−0.287	0.043
Anthropometric Measurements				
Height (m)	0.103	0.476	0.126	0.385
Weight (kg)	0.202	0.159	0.409	**0.003**
BMI (kg/m^2^)	0.191	0.185	0.380	**0.006**
Waist Circumference (cm)	0.233	0.120	0.421	**0.002**
Hip Circumference (cm)	0.137	0.343	0.373	**0.008**
Waist-to-Hip Ratio	0.254	0.075	0.371	**0.008**
BIA Measurements				
FM (kg)	0.112	0.440	0.315	0.026
Fat Mass (%)	−0.016	0.913	0.129	0.373
Visceral Fat (%)	0.320	0.024	0.463	0.001
Fat-Free Mass (%)	0.052	0.722	−0.094	0.514
Muscle Mass (%)	0.058	0.688	−0.088	0.542
Bone Mineral (%)	0.025	0.863	−0.126	0.384
Total Body Water (%)	0.051	0.727	−0.019	0.897
Nutritional Factors				
Glycemic Index	0.439	**0.001**	0.372	**0.008**
Energy (kcal/day)	0.214	0.136	0.368	**0.009**
Protein (g)	0.257	0.072	0.332	**0.018**
Protein (%)	0.147	0.309	0.105	0.466
Fat (g)	0.261	0.067	0.375	**0.007**
Fat (%)	0.228	0.111	0.202	0.160
Carbohydrate (g)	0.009	0.949	0.154	0.285
Carbohydrate (%)	−0.241	0.092	−0.225	0.117
Fiber (g)	0.194	0.177	0.229	0.110
Vitamin A	0.156	0.279	0.169	0.241
Vitamin B12	0.090	0.534	0.162	0.261
Vitamin C (mg)	−0.291	**0.040**	−0.309	**0.029**
Iron (mg)	0.192	0.182	0.256	0.073
Zinc (mg)	−0.338	**0.016**	−0.213	0.137
Adherence to Mediterranean Diet				
MEDAS Score	−0.580	0.000	−0.687	0.000
Daily Consumption of Specific Foods				
Cow Milk	0.166	0.249	0.092	0.526
Cheese	0.315	**0.026**	0.248	0.082
Yogurt	0.077	0.597	−0.096	0.508
Kefir	0.166	0.249	0.127	0.380
Dairy Products, Total *	0.233	0.103	0.055	0.706
Potatoes	0.108	0.454	0.152	0.292
Eggplants	0.183	0.204	0.105	0.469
Tomatoes	−0.173	0.230	−0.247	0.083
Peppers	−0.013	0.931	−0.021	0.884
Nightshade Vegetables, Total **	0.041	0.779	−0.004	0.980
Refined Sugar	0.581	**0.000**	0.580	**0.000**
Junk Foods	0.487	**0.000**	0.507	**0.000**
Bakery Products	0.296	**0.037**	0.410	**0.003**

A bolded *p*-value indicates a statistically significant difference (*p* < 0.05). * Dairy products, total: cow milk, cheese, yogurt, kefir. ** Nightshade vegetables, total: potatoes, tomatoes, peppers, eggplants.

**Table 6:** Factors potentially associated with disease severity.

IHS4						
Variable	Univariate (Beta)	Model R^2^	*p*-Value *	Multivariate (Beta)	Model R^2^	*p*-Value **
PAL	−19.583 (6.378)	0.164	**0.004** *	−7.357 (6.623)	0.500	0.273
MEDAS	−1.316 (0.201)	0.472	**0.000** *	−1.265 (0.287)		**0.000** *
GI	0.202 (0.073)	0.139	**0.008** *	0.059 (0.063)		0.352
BMI	0.214 (0.075)	0.145	**0.006** *	−0.084 (0.086)		0.335
**Hurley Stage**						
Variable	Univariate (Beta)	Model R^2^	*p*-Value *	Multivariate (Beta)	Model R^2^	*p*-Value **
PAL	−2.570 (1.538)	0.055	0.101	−0.719 (1.606)	0.428	0.657
MEDAS	−0.252 (0.051)	0.336	**0.000** *	−0.278 (0.070)		**0.000** *
GI	0.054 (0.016)	0.193	**0.001** *	0.027 (0.015)		0.083
BMI	0.024 (0.018)	0.036	0.185	−0.037 (0.021)		0.081

* *p*-value after using a simple linear regression model to compare the IHS4 score/Hurley stage and the other variables. ** *p*-value after using a multivariate regression model adjusted for PAL, MEDAS, glycemic index, and BMI. A bolded *p*-value indicates a statistically significant difference (*p* < 0.05).

## Data Availability

The data can be obtained from the corresponding author upon request.
